# Role of proteasomes in disease

**DOI:** 10.1186/1471-2091-8-S1-S3

**Published:** 2007-11-22

**Authors:** Burkhardt Dahlmann

**Affiliations:** 1 Institut für Biochemie, Charité-Universitätsmedizin-Berlin, Monbijoustr. 2, 10117 Berlin, Germany

## Abstract

A functional ubiquitin proteasome system is essential for all eukaryotic cells and therefore any alteration to its components has potential pathological consequences. Though the exact underlying mechanism is unclear, an age-related decrease in proteasome activity weakens cellular capacity to remove oxidatively modified proteins and favours the development of neurodegenerative and cardiac diseases. Up-regulation of proteasome activity is characteristic of muscle wasting conditions including sepsis, cachexia and uraemia, but may not be rate limiting. Meanwhile, enhanced presence of immunoproteasomes in aging brain and muscle tissue could reflect a persistent inflammatory defence and anti-stress mechanism, whereas in cancer cells, their down-regulation reflects a means by which to escape immune surveillance. Hence, induction of apoptosis by synthetic proteasome inhibitors is a potential treatment strategy for cancer, whereas for other diseases such as neurodegeneration, the use of proteasome-activating or -modulating compounds could be more effective.

**Publication history:** Republished from Current BioData's Targeted Proteins database (TPdb; ).

## Proteasome localization and function

As proteins play crucial roles in virtually all biological processes, the finely tuned equilibrium between their synthesis and degradation influences cellular homoeostasis. Protein degradation is predominantly catalysed by the proteasome, a giant protein breakdown enzyme complex, any disturbance to which can result in the onset of disease processes. The present review briefly summarizes some of the major aspects relating to the role of proteasomes in disease.

The majority of cellular proteins are degraded by the ubiquitin proteasome system (UPS), which consists of both substrate-recruiting and substrate-degrading machinery. The former is composed of three enzymes, the first of which (E1) activates the polypeptide ubiquitin in an ATP-dependent manner, enabling its transfer onto a ubiquitin carrier enzyme (E2). Activated ubiquitin is further transferred by a ubiquitin protein ligase (E3) to a substrate protein [1]. The substrate-recruiting machinery then catalyses the formation of an isopeptide bond between the C-terminal glycine residue of ubiquitin and the ε-amino group of a substrate protein lysine residue. Repeated addition of ubiquitin moieties onto the first results in a polyubiquitylated substrate protein that is recognized by the proteolytic machinery of the UPS, the 26S proteasome [2]. The 26S proteasome contains a central, barrel-like core particle (the 20S proteasome) composed of four stacked seven-membered rings, with the subunit stoichiometry α_1-7_β_1-7_β_1-7_α_1-7_[3]. A six-membered ring of AAA ATPase proteins binds to one or both outer α-rings and, together with two non-ATPase subunits, forms the base, while nine other subunits comprise the adjoining lid. In turn, the base and lid comprise the 19S regulator complex (19S REG) [4-6], which functions in the recognition of ubiquitylated substrates and their subsequent binding [7], de-ubiquitylation [8,9], unfolding and transfer into the central chamber of the 20S proteasome [10,11]. Within the 20S proteasome, subunits β1, β2, and β5 of both adjacent β-rings expose their proteolytically active sites, exhibiting post-glutamyl peptide hydrolysing (PGPH), trypsin-like and chymotrypsin-like cleavage specificity, respectively [12,13]. Under conditions of acute immune or stress response, these three β subunits can be substituted during *de novo* proteasome biosynthesis for the interferon-γ-inducible subunits β1i, β2i, and β5i. This results in the replacement of standard 20S proteasomes with immunoproteasomes, which have different cleavage specificities to those described above [14,15]. Replacement of only one or two of the active site-containing β subunits results in the formation of intermediate type proteasomes [16]. Alternatively, the proteasome activator PA28 can associate with the 20S proteasome in place of 19S REG [1].

In mammalian cells, proteasomes are located throughout the cytoplasm though most highly concentrated at the centrosome [18]. By contrast, immunoproteasomes specifically concentrate at the endoplasmic reticulum [19]. A nuclear localization signal directs proteasomes to enter the cell nucleus [20,21] (particularly after induction of cell stress [22]), in which they accumulate in focal subdomains [23]. Circulating 20S proteasomes (probably released from (dying) cells) have been found in human plasma and could potentially be used as diagnostic markers [24-26]. In yeast cells, the majority of proteasomes have been detected in the cell nucleus [27].

Whereas polyubiquitylation of proteins labels them for degradation by proteasomes, monoubiquitylation confers a cellular localization signal that, for example, directs them from the plasma membrane to endosomes and multivesicular bodies. The monoubiquitin localization signal also plays a role in regulating protein activity and function [28,29].

## The role of the proteasome in disease

By catalysing limited or complete degradation of proteins, the UPS functions in many basic cellular processes such as differentiation [30], proliferation [31], apoptosis [31], gene transcription [32], signal transduction [33], metabolic regulation [34], immune surveillance [35] and many others [36]. Thus, the UPS is essential for development and maintenance of all eukaryotic cells [37]. This biological importance implies that the UPS is also inevitably involved in patho-physiological processes resulting in the development of many diseases, including autoimmune, neurodegenerative and rheumatoid diseases, cancer, viral infections and cachexia (Table 1). Many of these pathological states are caused by defects in the E2 and E3 enzymes or by genomic or post-translational alterations to proteins that affect their ubiquitylation and subsequent susceptibility to proteasomal degradation. This in turn leads to disruptions in biochemical reaction sequences. Numerous reviews have summarized the UPS-associated patho-biochemical mechanisms of these disorders [e.g. [38-43].

**Table 1:** Adverse effects of the environment and aging on the proteasome system. The table summarizes diseases where proteasomes are known to be implicated and the impact that aging and environmental effectors could have on their development.

**Disease**	**Impairment of proteasomes**	**Proteasome population affected**	**Symptoms**	**References**
**Cardiac dysfunction**				
- transient ischemia/reperfusion	decreased activity	26S proteasomes	apoptosis	89, 90
- pressure overload	decreased activity	26S proteasomes	apoptosis	68
- inclusion body myositis	decreased activity	26S proteasomes, induction of immunoproteasome	inclusion bodies	83 134
**Cataract formation**	decreased activity	20S proteasomes	aggregation of (oxidized) proteins	66
**Neurodegenerative diseases**				
- Alzheimer's	decreased activity	20S/26S proteasomes	β-amyloid plaques/tau tangles, neuronal loss	77 78
- Parkinson's	decreased activity	20S/26S proteasomes	Lewy bodies, neuronal loss	73
- amyotrophic lateral sclerosis	decreased activity	20S/26S proteasomes	SOD1 aggregates, motor neuron loss	75
- Huntington's	decreased activity	20S/26S proteasomes, induction of immunoproteasome	poly-glutamine inclusions, neuronal dysfunction/loss	76 135
**Viral infections**				
- HIV/adenovirus	decreased expression, inhibition	immunoproteasomes, 20S proteasomes	impaired immune response	156 182
- hepatitis B	inhibition	20S/26S proteasomes	hepatitis	183
- HTLV	activation	nuclear proteasomes	neurological inflammation	184
**Autoimmune/rheumatoid diseases**				
- Sjogren's syndrome	decreased expression	subunit β1i	tissue destruction	185
**Cancer**				
- multiple myeloma - renal carcinoma	increased activity, depressed expression	20S/26S proteasomes, immunoproteasomes	suppression of apoptosis, induction of proliferaton	122 142,145
**Cachexia**				
- sepsis	increased activity	20S/26S proteasomes	Inflammation, muscle protein wasting	100
- metabolic acidosis	increased activity	20S/26S proteasomes	Inflammation, muscle protein wasting	97

The present paper summarizes knowledge of the changes in proteasomes that lead to the triggering and development of various diseases.

## Changes in proteasome activity – a cause of disease development?

In comparison to most other cellular proteins, the concentration of proteasomes is very high, ranging from 1 to 20 µg/mg of soluble protein [44]. As for all cellular proteins, proteasomes have a limited lifetime and thus are continuously synthesized and degraded. In HeLa cells, the half-life of standard- and immuno-proteasomes is around 5 days and 27 hours, respectively [14].

NLVS (4-hydroxy-5-iodo-3-nitrophenylacetyl-Ala-Ala-Phe-vinyl sulfone) is a compound that covalently binds the threonine residues of all three active site-containing β subunits, thus significantly reducing cellular proteasome activity. When mice thymoma and lymphoma cells are incubated with this compound, the residual proteasome activity (perhaps complemented by other proteases) ensures their survival. However, functions such as MHC class I antigen processing and presentation are clearly impaired by NLVS treatment [45]. This indicates that cells contain no real surplus of proteasome activity and that any change in the function of the complex could affect cellular homoeostasis. This may not necessarily compromise cell survival, but may be important enough to induce pathological consequences, for example, through impaired activation of the transcription factor NFκB, which mediates cellular responses to the many signals received from outside [46].

## Decreased proteasomal activity and disease

An age-related decrease in proteasome activity has been observed in different tissues including bovine eye lens [47], rat liver [48,49], human skin and epidermal cells [50,51], rat heart [52], human lymphocytes [53], human fibroblasts [54], rat kidney [55], rat lung [55] and rat muscle [56,57]. In all of these investigations, proteasome activity was measured using fluorigenic peptide substrates and PGPH activity was found to be consistently depressed with increasing age. Only in rat liver extracts [48] was an increase in chymotrypsin- and trypsin-like activity observed (when measured in the presence of SDS) with increasing age. However, when specific activity was measured in purified liver 20S proteasomes, a loss of PGPH activity was observed with no change in chymotrypsin- and trypsin-like activity [49]. Therefore, results obtained by use of tissue extracts [48,50,52] may not reflect the intrinsic activity of proteasomes, but in fact be influenced by other components within the homogenate, for example proteasome activator PA28 or 19S REG [57]. Alternatively, decreased activity could be due to reduced amounts of proteasome, as observed in aging keratinocytes [50], but not in liver tissue of aged rats when compared with young rats [48]. However, when the specific activity of 20S proteasomes purified from rat liver [49], rat heart [52], human epidermis [51] and bovine lens [47] was calculated, a loss of PGPH activity [49] or PGPH and chymotrypsin-like activity [51], or PGPH and trypsin-like activity [47,52] was detected, supporting the link between intrinsic changes in 20S proteasomes and increasing age. In some of the studies mentioned [49-52], a rise in the concentration of oxidatively modified proteins was detected in aged donor tissue samples. Since oxidatively modified proteins have been found to be substrates of 20S proteasomes [58], all of these investigations have been confined to 20S proteasomes. The concentration of ubiquitylated proteins was also found to be increased in aged tissue and cells [52,53], suggesting that the activity of 26S proteasomes could be affected by aging in addition to 20S proteasomes. Specifically, this was shown in human lymphocytes of elderly donors [53], as well as in late passages of a human fibroblast cell line, which both exhibited a significant reduction in 26S proteasome activity [54]. Accordingly, a cell model for studying aging processes has been developed by Chondrogianni and Gonos (Institute of Biological Research and Biotechnology, Athens, Greece) that uses human embryonic lung fibroblasts in which a senescence-like phenotype is induced by treatment with low doses of commercially available proteasome inhibitors (epoxomicin or MG132) [59]. Epoxomicin, a microbial epoxyketone, is known to react irreversibly with all three active sites but primarily with the β5 subunit, which exhibits chymotrypsin-like activity [60]. Though less specific, MG132, a peptide aldehyde (Z-L-leucyl-L-leucyl-L-leucinal), is probably the most widely used proteasome inhibitor. It reacts reversibly, primarily with proteasome subunit β5 [61].

As aging is a progressive and irreversible (but not pathological) phenomenon, a decline in proteasome activity may be regarded as the natural answer to an age-related decrease in the rate of protein synthesis, a process counterbalanced by protein degradation. Therefore, it is no surprise that experimental inhibition of proteasome activity in primary neuronal cells, for example, induces impairments in protein synthesis [62], especially since many transcription factors and some translation-initiation factors, as well as ribosome biogenesis, are regulated by proteasomal processing [63-65]. However, as previously mentioned, proteasome activity may also be necessary to remove products of other processes that become imbalanced during aging, such as the antioxidant response system. For example, a lowered capacity to eliminate oxidatively damaged proteins due to a decrease in all three proteasome peptidase activities in eye lens nuclei favours cataract formation in elderly individuals [66] and, in heart muscle, reduces tolerance of the aged heart to ischemia/reperfusion [67]. Experimental thoracic aortic constriction was also found to depress proteasome activity in mouse heart and thus favours accumulation of pro-apoptotic proteins that later induce apoptosis and cardiac dysfunction [68].

Using fluorigenic peptide substrates, an age-related decrease in 20S proteasome activity has been detected in certain areas of rat, mice and marmoset brain. These especially include the substantia nigra, striatum, cerebral cortex and spinal cord but not cerebellum [55,69,70], although statistical significance of the difference in activity could depend on the interval of age chosen for the investigations [71]. A life-long steady decrease in proteasome activity is proposed to be responsible for accumulation of abnormally folded proteins, formation of inclusion bodies and development of neurodegenerative diseases such as Alzheimer's and Parkinson's disease, amyotrophic lateral sclerosis and Huntington's disease [72-76].

Thus, age-related proteasomal dysfunction could be regarded as a factor in these disease processes, which involve the formation of plaques, filaments and aggregates. Once generated, these protein inclusions have been found to further inhibit proteasome activity and thus amplify the formation of inclusion bodies [77-80]. Additionally, the age-related loss of antioxidant capacity affects the proteasomal system, particularly the 26S proteasome, which appears to be itself sensitive to oxidative inactivation [81]. The latter finding has been tested by measuring proteasome activity in the presence and absence of ATP in neuronal cells exposed to non-toxic doses of hydrogen peroxide (H_2_O_2_) [82]. In either case, residual proteasomal capacity appears unable to eliminate the aggregated and highly oxidized proteins [83], leading to irreversible development of neurodegenerative diseases.

## Which mechanisms underlie the age-related attenuation of proteasome activity?

Investigations into the mechanisms underlying the change in proteasome activity during aging are usually performed by comparing cells and tissues from individuals (mostly rats) of different ages, since experimental models mimicking the aging process are difficult to obtain. In particular, the effect of aging on proteasome activity cannot be mimicked by proteasome knockout models, as they are non-viable [37].

Therefore, data summarized in this review were obtained from studies utilising many different tissues from various species. In most investigations into age-dependent changes in proteasome activity, a decrease in PGPH activity was detected, while changes in chymotrypsin-like and trypsin-like activity were not as consistent. Thus, a general decrease in 20S/26S proteasome concentration, as found in aging keratinocytes [50] and in a human fibroblast cell line that was experimentally induced to senescence-like phenotype by incubation with low dose proteasome inhibitors [54], cannot be the general underlying mechanism. Certainly, a lower concentration of subunit α4 mRNA was measured in mesencephalon of adult rats when compared with young, suggesting an age-dependent decreased expression of proteasomes [84]. However, if this were the case, a general decrease in all three proteasome activities catalyzed by subunits β1 (PGPH-activity, β2 (trypsin-like activity) and β5 (chymotrypsin-like activity) should be observed during aging.

Glycation and/or conjugation with the lipid peroxidation product 4-hydroxy-2-nonenal of several α and β subunits (including active site-containing β subunits) was found to coincide with a decrease in chymotrypsin-like activity of 26S proteasomes in lymphocytes from healthy 50–63 year old human donors when compared with 20–35 year old donors [53]. Only two subunits from the 19S REG base were glycated without an effect on the stability and abundance of 26S proteasomes [53]. This observation corroborates the finding that aging does not coincide with an enhanced dissociation of 26S proteasomes into core 20S particles and 19S regulators [54]. Similarly, modifications by 4-hydroxy-2-nonenal, a peroxidation product derived from oxidized ω-6 polyunsaturated fatty acids [85], were found to affect neuronal proteasomes [86-88], as well as myocardial proteasomes after coronary occlusion/reperfusion [89,90].

The chaperone Hsp90 was shown to protect 20S proteasomes from oxidative inactivation in rat hepatoma cells treated with iron/ascorbate [91]. Interestingly, 20S proteasomes were found to be associated with Hsp90 when purified from bovine lens of one month old but not of two year old animals [47]. This suggests an age-dependent loss of the protective agent in bovine lens, thus favouring oxidation of proteins including proteasomes [92], leading to age-related cataract formation [93]. Similar to the proteasome, Hsp90 is essential for cell survival and therefore this protecting function cannot be tested with Hsp90 knockout cells.

Complex alterations in the proteasome system were found in sarcopenia of rat soleus muscle. Specifically, the concentration of 20S proteasomes in the muscle of old rats (29–40 months) was three-fold higher, but of significantly lower specific activity towards fluorigenic peptide substrates, when compared with young rats (5–12 months) [94]. This loss of activity may in part be due to oxidative modification because it was partly rescued by addition of the reducing agent dithiothreitol (DTT) to the test system [95]. The three-fold increase in 20S proteasome concentration led to increased ratios of 20S proteasomes to both PA28 proteasome activator and 19S REG in aged rats when compared with young rats. Additionally, the interferon-γ-inducible proteasome subunits β1i and β5i were detected in 3–6 fold higher amounts in aged muscle compared with young muscle. Interestingly, high expression of β1i and β5i was also found in neurons, astrocytes and endothelial cells of the hippocampus region of elderly humans (mean age 70 years), but only scarcely in that of young donors (mean age 42 years) [96]. These data suggest that during aging, intermediate-type proteasomes and immunoproteasomes may accumulate in muscle and brain, tissues that normally predominantly contain standard proteasomes.

## Enhanced proteasome activity and disease

Muscle atrophy due to decreased protein synthesis and accelerated protein degradation is a hallmark of many patho-physiological situations, examples of which are chronic kidney diseases, type I diabetes mellitus, sepsis, cancer cachexia and starvation. Increased expression of proteasomes in muscle tissue has been observed in rats suffering from NH_4_Cl-induced metabolic acidosis [97], tumors [98], starvation, denervation atrophy [99], sepsis induced by cecal ligation and puncture [100] and other catabolic conditions [reviewed in [101]], as shown by enhanced transcription of genes encoding proteasome subunits and enhanced proteasomal activity towards fluorigenic substrate proteins [100]. mRNA levels of the 19S REG subunit Rpt1 were also found to be increased in starving rat skeletal muscle; however, knowledge of the adaptive mechanisms of this 26S proteasome sub-complex to catabolic situations is still very limited [102]. Accelerated muscle protein breakdown in burn-injured rats can be inhibited by the proteasome inhibitor lactacystin and also by the less specific but commercially available reversible proteasome inhibitor LLnL (*N*-acetyl-L-leucyl-L-leucyl-L-norleucinal) [103], which binds to all three active sites with different affinities [104]. The same inhibitors [105], as well as the commercially available proteasome inhibitor PSI (*N*-benzyloxycarbonyl-Ile-Glu-(*O*-t-butyl)-Ala-leucinal), which blocks the chymotrypsin-like activity of proteasomes [106], prevent the sepsis-induced increase in protein degradation in rat skeletal muscle [107]. These data clearly demonstrate that proteasomes are responsible for enhanced muscle protein degradation under catabolic conditions [108,109]. However, proteasomes appear not to be the initiating enzymes under these conditions, since they are not able to degrade contractile proteins and their regulators while they are assembled within the myofibrils that constitute the majority of muscle proteins [110]. Rather, the protease caspase-3, the activity and amount of which is increased in rat skeletal muscle under experimental diabetes mellitus, appears to catalyse the disassembly of myofibrils [111,112]. This leads to the delivery of substrates to muscle-specific E3 ubiquitin protein ligases (such as atrogin-1 and MuRF1), which prepare muscle proteins for subsequent proteasomal degradation [113]. Using microarray hybridization, the mRNAs of both E3 ligases (similar to that of several proteasome subunits) were found to be expressed at a significantly higher level in mice and rat skeletal muscle during fasting, tumor bearing, chronic renal failure and experimentally induced diabetes mellitus [114]. This suggests that in some cases, compounds inhibiting caspase-3 and E3 enzymes could be more useful in the clinical management of these muscle wasting conditions than proteasome inhibitors. However, a diminution of proteasomal activity could still be useful, for example in preventing degradation of IκB and thus activation of NFκB, since this transcription factor appears to be involved in the induction of muscle atrophy [115] by possibly up-regulating MuRF1 [116]. These findings corroborate the data referred to earlier, which showed that treatment of rats with proteasome inhibitors suppresses their muscle atrophy induced by sepsis or burn injury [103,105,106].

A complex role for the proteasome in apoptosis is supported by the finding that inhibition of proteasome activity has pro- as well as anti-apoptotic effects [31]. The activation of NFκB by proteasomes (through degradation of IκB) induces the expression of anti-apoptotic members of the BCL2 family that maintain the mitochondrial membrane barrier [117]. Additionally, proteasomes degrade pro-apoptotic proteins such as Bax and Bid [118,119], the tumor suppressor p53 [120] and the negative cell cycle regulators p21 (Cip1) and p27 (Kip1) [reviewed in [121]]. All of these anti-apoptotic proteasomal effects are predominantly found in neoplastic and rapidly growing cells. Therefore, a main treatment strategy for multiple myeloma involves the induction of apoptosis through the introduction of proteasome inhibitors such as bortezomib and NPI-0052 into these cells [122,123].

Bortezomib (formally known as MLN341, PS-341, LDP-341) is a dipeptide boronate (pyrazylcarbonyl-phenylalanyl-leucyl-boronate) originally developed at ProScript and now distributed by Millennium Pharmaceuticals. It binds to the N-terminal threonine hydroxyl group of the active site-containing β5 subunit in a similar manner to peptide aldehyde inhibitors but with a slower dissociation rate and higher specificity, since no protease other than the proteasome was found to be inhibited in rat [124]. As this proteasome inhibitor was found to be active against a broad range of tumours, it entered clinical trials and is now used as Velcade^®^ in clinical oncology for treatment of multiple myeloma. NPI-0052 (Salinsporamide-A) is a non-peptide proteasome inhibitor from the marine actinomycete *Salinospora* containing a β-lacton structure similar to that of lactacystin. It was found to irreversibly inhibit all three proteasomal peptide cleaving activities. This compound is being developed by Nereus Pharmaceuticals and is currently in Phase I clinical trials for the patients resistant to Velcade^®^ treatment [125].

Referring back to the complex role of proteasomes in apoptosis, a clear pro-apoptotic proteasomal activity was observed in human umbilical vein endothelial cells (HUVEC), primary thymocytes and neurons. Specifically, BCL2 and inhibitors of apoptosis proteins (IAP) were shown to be degraded by proteasomes, resulting in a stimulation of apoptosis that could be prevented by treatment of these cells with the proteasome inhibitors MG132 (*Z*-L-leucyl-L-leucyl-L-leucinal), ALLN (Acetyl-L-Leucyl-L-Leucyl-L-Norleucinal) or lactacystin [126-128]. MG132 is more potent and more selective than ALLN, which was originally used as an inhibitor of calpains, a heterogenous family of Ca-dependent cysteine proteinases [129]. These data indicate that therapeutic use of proteasome inhibitors must therefore be based on a clear knowledge of the underlying molecular mechanism of the disease.

Furthermore, elevated proteolytic and ATPase activity due to increased expression of 20S proteasome and 19S REG ATPase subunits was found to occur during developmentally regulated and endogenously triggered apoptosis [130-132].

## Immunoproteasomes and disease

Transcription of the gene encoding the immunoproteasome subunit β1i requires binding of the transcription factors Stat-1 and IRF-1 to the partially overlapping interferon-consensus-sequence-2/γ-interferon-activated sequence (ICS2/GAS) in its promoter region. Due to the presence of interferon-stimulated response elements, transcription and expression of β1i (as well as β5i) is induced by γ-interferon. However, cells professionally involved in immune surveillance, such as splenocytes and dendritic cells, constitutively express immunoproteasomes due to binding of unphosphorylated and non-dimerized Stat-1 to ICS2/GAS [133]. If, as mentioned above, brain and muscle tissue show an age-related increase in the concentration of immunoproteasome subunits, it could reflect a state of constant inflammation or cell stress. Thus, increased concentration of immunoproteasomes in muscle of patients suffering from myofibrillar myopathy and inclusion body myositis [134], as well as in neurons of a mouse model of Huntington's disease [135], can be regarded as a consequence of, rather than the cause of, these diseases. Alternatively, this increased concentration could result from a potential defence mechanism, since induction of immunoproteasome subunits was recently shown to occur in endothelial cells by nitric oxide (NO) via cGMP/cAMP-mediated mechanisms. The NO-induced synthesis of immunoproteasomes protected the cells against transferrin iron-induced oxidative stress by regulating the level of transferrin receptor [136]. Since NO regulates processes such as vasodilatation, neurotransmittance, the immune response and apoptosis, an imbalance in this mediator has many pathological consequences. An NO-dependent change in the ratio of standard- to immuno-proteasomes is thought to contribute to these consequences [137-140]. Similarly, treatment of SH-SY5Y neural cells with non-toxic doses (1–10 µM) of H_2_O_2_ induced not only the formation of oxidized proteins but also synthesis of immunoproteasome subunits (detected by western blotting and real-time RT-PCR) [82], indicating the sensitivity of the proteasome system to react and to cope with cell stress. Such an adaptation of the proteasome system was found to be lost in senescent human fibroblasts, which displayed a decreased concentration in standard proteasome subunits but retention in the immunoproteasome subunits β1i, β2i and β5i. In contrast to confluent young fibroblasts, the concentration of immunoproteasome subunits could not be augmented by treatment with γ-interferon [141].

The adverse process, namely down-regulation of immunoproteasomes, has been found to serve as an immune surveillance escape mechanism in several tumor cells [142-145], since generation of certain MHC class I-presented antigenic epitopes appears less efficient when performed by standard proteasomes compared with immunoproteasomes. Another successful oncogenic mechanism relying on insufficient antigen processing was observed in human cervical carcinoma and melanoma cells that express a non-functional variant of the immunoproteasome subunit β5i, designated E1. In contrast to its functional counterpart, designated β5i-E2, variant E1 cannot be recruited and incorporated by the proteasome maturation factor, POMP, into nascent 20S proteasomes [146]. This defect prevents formation of immunoproteasomes and suppresses the generation of epitopes that elicit a cytotoxic immune response. It is therefore not surprising that the human immunodeficiency virus (HIV) interferes with the antigen processing/presentation machinery of its host cell by means of competition between HIV-tat protein and Stat-1 for binding to IRF-1, thus suppressing synthesis of β2i and functional immunoproteasomes [147].

## Disease targets and ligands

The three different proteolytically active sites of the 20S proteasome use the same catalytic mechanism in which the N-terminal threonine residue is the active nucleophile. Therefore, many proteasome inhibitors that modify this threonine residue, for example aldehydes [148], epoxyketones [149], vinyl sulfones [150], lactacystin [151] and boronates [152], affect all three peptide bond cleaving activities, though to differing extents due to different binding affinities within the substrate binding pockets and mostly with a preference for chymotrypsin-like activity [153]. By using more ‘site-specific’ inhibitors, Kisselev *et al.*[154] found that the relative importance of each active site for the degradative process depends on the individual substrate. Thus, site-specific inhibitors, as long as they are membrane permeable, could be helpful in treating malignant neoplastic diseases other than multiple myeloma, since proteasome-dependent processes such as neovascularization, cell adhesion and intravasation might be more sensitive to this type of inhibitor. Site-specific proteasome inhibitors could also be especially useful as immunosuppressive agents in autoimmune diseases by modulating antigen processing and MHC class I-restricted antigen presentation, and in retroviral infections [155,156]. For similar purposes, compounds that specifically inhibit immunoproteasomes are conceivable [157].

In addition to the ability to permeate biological membranes, proteasome-inhibiting compounds should also exhibit low toxicity. A lead compound for the development of non-toxic compounds could be the naturally existing, commercially available proteasome inhibitor PR39, a 39 amino acid peptide rich in proline and arginine residues with antimicrobial activity. This peptide was shown to allosterically inhibit the chymotrypsin- and caspase-like activities of 20S proteasomes. In addition, the N-terminal 11mer fragment of PR39, known as PR11, was also shown to display inhibitory activity [158]. PR39 attenuates post-ischemic microvascular injuries and inflammatory reactions [159,160] and appears to bind to the outer α-ring of 20S proteasomes [161].

Originally, proteasome inhibitors such as bortezomib (at the time still designated MG341) were also tested in animal models for treatment of acute and chronic inflammatory reactions, for example delayed type hypersensitivity and arthritis. Oral doses of the inhibitor effectively cured or even suppressed allergic and inflammatory reactions [162]. As proteasomes are involved in key signalling pathways regulating inflammation and sepsis [163,164], the design of new proteasome inhibitors aimed at these important targets would be useful in the treatment of related diseases.

In contrast to the use of proteasome inhibitors, there are many pathological situations characterized by a depressed proteasomal proteolytic activity that could be ameliorated by proteasome-activating compounds, an area of the field that has received little attention to-date [165]. Although the exact mechanism of the age-related decrease in proteasome activity is unknown, proteasome-activating hydrophobic peptides have been found that most probably bind as modifiers at non-catalytic sites, thus mimicking the effect of the proteasome activator PA28 by opening the gated α-ring pore [166]. These peptides and other compounds that were shown to activate 20S proteasomes such as fatty acids, phosphatidyl-glycerol and di-phosphatidyl-glycerol [167], could be used as starting points for generating proteasome activating compounds.

## Next Frontiers

Although a great deal has been published about changes in proteasome activity and their possible involvement in the development of diseases, there are clear gaps in our knowledge of the definite molecular mechanisms that underlie these alterations. This holds especially true with regard to the fact that proteasomes in various cells and tissues are not a multitude of identical 20S proteasomes but a mixture of several proteasome subpopulations (standard- and immuno-proteasomes) and intermediates of each subpopulation [16,168], which have different substrate specificities [16]. The distribution within the different cell compartments of these subpopulations and the proportions of the various proteasome complexes, for example 26S proteasomes, hybrid proteasomes and proteasome–PA28 complexes are only scarcely known. Additionally, knowledge of the molecular differences between the various proteasome subtypes, which appear to have different susceptibilities to inhibitors [A. Kloß and B. Dahlmann, unpublished observation] is only superficial [169]. These aspects, along with post-translational modifications such as phosphorylation, O-GlcNAc addition and others [170,171] that affect proteasome activity, need to be investigated intensively with regard to disease-related alterations to the proteasome system. Since the proteasome is considered to be a therapeutic target, this knowledge will also help to design more specific and probably more effective compounds that modulate its activity.

It is clear that modulation of proteasome activity is primarily aimed at inhibiting the complex's proteolytic functions. However, the finding that the association between 19S REG and 20S proteasomes is regulated by a proteasomal ATPase-associated factor provides another promising aspect to explore in the search for compounds that modulate proteasome activity [172]. To mimic or inhibit the activity of this factor might help to tune the activity of 20S/26S proteasomes without risking a complete shut off of this essential system. Similarly, compounds modulating the ATPase activity of the 19S regulator, as well as its polyubiquitin-binding ability, could be considered for therapeutic use.

A completely different aspect for future research with regard to the benefit of proteasome inhibitors is based on the finding that inhibition of proteasomal activity induces *de novo* synthesis of proteasomes [173]. This knowledge may be applicable to situations where disease processes appear to be based on reduced amounts of, or defects in, proteasomes, for example as occur during aging. Similarly, treatment of endothelial cells with low, non-toxic doses of the proteasome inhibitor MG132 was found to activate an antioxidant defence programme that included up-regulation of endothelial nitric oxide synthase (eNOS), glutathione peroxidase-3, glutathione S-transferase and others, resulting in an improvement in endothelial functions [174,175]. Recognition of this effect is not only promising in the search for treatments for patients suffering from atherosclerosis and coronary heart diseases, but also to provide a possible measure for preventing these diseases and others such as neurodegeneration [176]. Based on the same biochemical background, vascular endothelial dysfunction and neuronal death after embolic stroke were found to be restricted and terminable in animal models after proteasome inhibitors such as bortezomib [177,178] or MLN519 [179] were administered within a certain therapeutic window of a few hours post-stroke*.* MLN519 ((1*R*-[1*S*,4*R*,5*S*])-1-(1hydroxy-2-methylpropyl-6-oxa-2-azabi-cyclo [3.2.1] heptane-3,7-dione) is a synthetic analog of lactacystin developed by Millennium Pharmaceuticals and already tested in phase I clinical trials [180]. An exact knowledge of the susceptibility of the different forms of proteasomes to this and other inhibitors will help to develop optimal treatments and therapies for a wide range of diseases.

Finally, aside from proteasome inhibitors or activators, proteasomes *per se* could be useful as diagnostic and even prognostic markers, since the level of circulating proteasomes reflects the state of health of patients suffering from cancer and autoimmune diseases [181].

## Abbreviations used

Hsp90, heat shock protein 90; HIV, human immunodeficiency virus; HTLV, human T-cell leukaemia virus; PGPH, post-glutamyl peptide hydrolysing; Ub, ubiquitin; UPS, ubiquitin proteasome system; 19S REG, 19S regulator.

## Competing interests

The authors declare that they have no competing interests.

## Publication history

Republished from Current BioData's Targeted Proteins database (TPdb; ).
